# Characteristics Associated with Biologic Monotherapy Use in Biologic-Naive Patients with Rheumatoid Arthritis in a US Registry Population

**DOI:** 10.1007/s40744-015-0008-9

**Published:** 2015-01-27

**Authors:** Dimitrios A. Pappas, George W. Reed, Katherine Saunders, Ani John, Ashwini Shewade, Jeffrey D. Greenberg, Joel M. Kremer

**Affiliations:** 1 New York—Presbyterian Hospital/Columbia University Medical Center, 630 West 168th Street, P&S Building, 10th Floor, New York, NY 10032 USA; 2 Corrona, LLC, 352 Turnpike Rd, Suite 325, Southborough, MA 01772 USA; 3 Genentech, Inc, 1 DNA Way, South San Francisco, CA 94080 USA; 4 New York University School of Medicine, 550 1st Ave, New York, NY 10016 USA; 5 Albany Medical College and The Center for Rheumatology, 1367 Washington Ave, Suite 101, Albany, NY 12206 USA

**Keywords:** Biologic agents, Biologic monotherapy, Disease-modifying antirheumatic drugs, Prescribing patterns, Registry, Rheumatoid arthritis

## Abstract

**Introduction:**

The aim of this study was to describe factors associated with initiating a biologic as monotherapy vs in combination with a conventional disease-modifying antirheumatic drug (DMARD) in biologic-naive patients with rheumatoid arthritis (RA) enrolled in the Corrona registry.

**Methods:**

First biologic initiations were classified as monotherapy (Bio MT) or combination therapy (Bio CMB). Baseline demographic and clinical characteristics were evaluated. Odds ratios (OR) based on mixed effects regression models estimated the association of covariates and use of monotherapy. Median odds ratios (MOR) based on estimated physician random effects quantified variation in individual physician use of monotherapy.

**Results:**

Between October 2001 and April 2012, 3,923 previously biologic-naive patients initiated biologic therapy, of which 19.1 % initiated as monotherapy. Baseline characteristics of patients initiating Bio MT and Bio CMB were similar for age, sex, duration of RA, and clinical disease activity index. Significantly higher proportions of Bio CMB initiators had prior conventional DMARD (97.23 vs 85.60 %; *P* < 0.01) and methotrexate (MTX) use (91.68 vs 71.87 %; *P* < 0.01) compared with Bio MT initiators. Variation in individual physician use of monotherapy [MOR 1.89; 95 % confidence interval (CI), 1.66–2.23] and use of biologics approved by the United States Food and Drug Administration for monotherapy (OR 1.47; 95 % CI, 1.20–1.81) significantly influenced the odds of initiating Bio MT. Patient history of hepatic disease, neutropenia, and malignancy were associated with increased odds of being prescribed Bio MT.

**Conclusion:**

In addition to regulatory approval for monotherapy and specific pre-existing comorbidities, significant variation in physician use of monotherapy was associated with increased likelihood of initiating Bio MT, independent of patient factors.

**Electronic supplementary material:**

The online version of this article (doi:10.1007/s40744-015-0008-9) contains supplementary material, which is available to authorized users.

## Introduction

Rheumatoid arthritis (RA) is a chronic, systemic autoimmune disease manifesting as joint inflammation that, if left untreated, eventually leads to joint damage, destruction, and disability. International task forces recommend conventional disease-modifying antirheumatic drugs (DMARDs) as first-line therapy in patients with RA, which should be started as soon as the diagnosis has been made with the goal of achieving remission or low disease activity [1, 2]. However, a proportion of patients fail to respond to conventional DMARDs. In addition, a number of patients may not be able to tolerate conventional DMARDs due to medication toxicity, contraindicating comorbidities, or interactions with other medications [3]. In such patients, treatment with a biologic DMARD as monotherapy may provide clinical benefit while sparing the patient from undesirable side effects due to conventional DMARDs [4].

Five classes of target-specific biologic DMARDs are currently available for patients with RA who do not respond or cannot tolerate conventional DMARDs. These include anti–tumor necrosis factor (TNF) agents (adalimumab, certolizumab pegol, etanercept, golimumab, and infliximab), an anti-interleukin (IL)-6 receptor antibody (tocilizumab), an anti-CD20 antibody (rituximab), an IL-1 receptor antagonist (anakinra), and a T cell costimulation modulator (abatacept). In the United States and Europe, most biologics—with the exception of rituximab, infliximab, and golimumab—are approved for use as monotherapy in patients with RA. In addition, the oral small molecule Janus kinase inhibitor (tofacitinib) may be used as monotherapy or in combination with methotrexate (MTX) or other conventional DMARDs.

Numerous studies of patients who have an inadequate response to DMARDs have demonstrated that biologic agents, such as anti-TNFs, offer higher levels of disease control, better symptomatic improvement, and possibly improved prevention of radiographic progression when prescribed in combination with MTX than when prescribed as monotherapy [5–8]. On the other hand, in a single study of patients with an inadequate response to MTX, tocilizumab demonstrated similar clinical efficacy when prescribed as either monotherapy or in combination with MTX [9]. In addition, real-world data derived from registries and claims database studies in the United States and Europe have reported that 12–39 % of patients with RA receiving biologics take them as monotherapy [3, 10–16].

The factors that influence physicians to prescribe biologic monotherapy, as opposed to biologics in combination with DMARDs, in routine clinical practice may be complex and have not been thoroughly evaluated. In a retrospective cohort study, the most common reasons for prescribing biologic monotherapy (anti-TNFs or tocilizumab) were intolerance to MTX, presence of contraindications to this agent or comorbidities, discontinuation of DMARDs due to lack of biologic efficacy, or patient preference [17].

The aim of this study was to further investigate the factors that may influence the decision to start a biologic as monotherapy or in combination with conventional DMARDs in a real-world cohort of biologic-naive patients with RA.

## Methods

### Study Population

The Corrona registry is an independent, prospective observational cohort of patients with RA recruited at more than 160 private and academic practice sites across 40 states in the United States, with more than 600 participating rheumatologists. As of March 31, 2014, data on approximately 39,950 patients with RA have been collected. Corrona’s database includes information about 285,726 patient visits and approximately 119,298 patient-years of follow-up observation time, with a mean time of patient follow-up of 3.6 years (median, 2.8 years). Details of the Corrona registry design have been previously described [18].

At each Corrona registry visit, patients and physicians record data on disease severity and activity, RA and other medications, adverse events, quality of life, selected laboratory and imaging results, and socio-demographic information. For this study, patients with RA who had previously received only conventional DMARDs and were initiating their first biologic were included in the analysis.

All patients in Corrona had previously provided written informed consent for participation in the registry. The Corrona protocol was approved by the institutional review boards of participating academic sites and a central institutional review board for private practice sites.

### Statistical Analysis

Every biologic initiation was categorized as monotherapy (Bio MT) or in combination with a conventional DMARD (Bio CMB). Baseline characteristics were compared between patients receiving Bio MT and those receiving Bio CMB, and included patient demographics, disease characteristics, concurrent medications, and history of comorbidities. In addition, reasons for discontinuation of previously administered conventional DMARDs were described for patients initiating Bio MT and Bio CMB. Demographic and educational characteristics of prescribing physicians were also summarized.

Mixed effects logistic regression models were estimated to examine predictors of monotherapy in biologic-naive initiators with the prescribing physician as a random effect. The random effect accounted for the correlation of treatment decision (monotherapy or combination therapy) among patients treated by the same physician. Potential covariates for the multivariable models included factors based on biologic plausibility in addition to any patient or physician covariates that were significantly different between Bio MT and Bio CMB initiations in univariate comparisons (*P* < 0.05). Whether the biologic initiation took place before or after the year 2006, when more biologics became available and/or approved for monotherapy, was included in the models to adjust for confounding factors. After the initial subset of significant covariates was determined, covariates that were not significantly different were considered for addition into the model but were not significantly associated. Additionally, covariates (such as presence of joint erosions or low neutrophil counts) that were significantly different in univariate comparisons between Bio MT and Bio CMB, but resulted in a reduction of sample size due to data availability, were considered in separate reduced sample models to illustrate their potential impact as sensitivity analyses. As disease activity measures are collinear, the choice of the measures used in the regression models was determined using both Akaike information criteria and Bayes information criteria [19, 20].

The estimated physician random effects measure the variation in rates of monotherapy among physicians due to unmeasured heterogeneity among physicians’ patient populations and in physicians’ treating patterns. A median odds ratio (MOR) was computed as a measure to compare the impact of variation in individual physicians’ use of biologic agents as monotherapy compared with the fixed effects in the model [21]. An odds ratio (OR) was calculated for all possible pairs of physicians’ rates of Bio MT, resulting in a distribution of ORs for physicians from highest rate to lowest rate of Bio MT prescription. The median of this distribution was designated as the MOR. The MOR can be directly computed from the variance of the random effects and the 95 % confidence interval (CI) was calculated from the 95 % CI of the variance. All statistical analysis was completed using STATA, version 12.1 (StataCorp LP, College Station, TX).

## Results

### Patient Disposition and Baseline Characteristics

Between October 2001 and April 2012, a total of 3,923 biologic-naive patients with RA initiated a biologic agent. The biologic agent was initiated as monotherapy in 750 patients (19.1 %) and in combination with conventional DMARDs in 3,173 patients (80.9 %). Baseline demographics and disease characteristics of patients receiving Bio MT and Bio CMB are shown in Table 1. Patients who initiated Bio MT or Bio CMB were similar with respect to age, sex, duration of disease, and clinical disease activity index at baseline (Table 1). Of patients who received Bio CMB, the majority (83.9 %) received concurrent MTX (Table 1). Patients who initiated Bio CMB had significantly more joint erosions, higher swollen joint counts, and greater likelihood of prior MTX and DMARD use compared with patients who initiated Bio MT. In contrast, a significantly higher proportion of patients receiving Bio MT had a history of cancer, hepatic events, and neutropenia compared with patients initiating Bio CMB. The rate of Bio MT initiation was similar before and after 2006 [19.7 % (*n* = 270/1,369) vs 18.8 % (*n* = 480/2,554), respectively], regardless of the increased availability of monotherapy options after 2006.

**Table 1:** Patient demographics and clinical characteristics at time of biologic initiation

	Biologic-naive patients (*N* = 3,923)	Initiated Bio MT (*n* = 750)	Initiated Bio CMB (*n* = 3,173)	*P* value^a^
Age, mean (SD), years	57.33 (13.5)	56.85 (14.7)	57.45 (13.2)	0.28
Female, %	75.86	74.42	76.19	0.32
White, %	81.55	83.56	81.08	0.13
Duration of RA, mean (SD), years	8.28 (9.3)	8.47 (9.0)	8.24 (9.3)	0.54
RF seropositivity, %	75.43	75.43	75.43	1.00
Disease activity, mean (SD)				
Tender joints, 28 count	6.05 (6.8)	5.98 (7.0)	6.07 (6.8)	0.75
Swollen joints, 28 count	6.15 (6.2)	5.33 (6.0)	6.34 (6.2)	<0.01
Physician global assessment	31.87 (22.1)	31.02 (22.6)	32.07 (22.0)	0.24
Patient global assessment	38.63 (27.2)	39.85 (29.1)	38.35 (26.7)	0.19
Patient pain	41.44 (29.1)	43.22 (35.3)	41.02 (27.5)	0.07
mHAQ score, mean (SD)	0.47 (0.5)	0.47 (0.5)	0.47 (0.5)	0.90
CDAI score, mean (SD)	19.27 (14.0)	18.57 (14.3)	19.43 (13.9)	0.14
Erosive disease, %	46.63	41.93	47.73	0.02
Current smoker, %	19.69	20.50	19.50	0.57
Comorbidities, %				
History of MI	2.70	2.93	2.65	0.62
History of stroke	1.99	2.27	1.92	0.56
History of CVD	0.69	0.40	0.76	0.46
History of cancer	0.74	1.47	0.57	0.02
History of serious infections	1.02	1.52	0.89	0.27
History of hepatic events	0.99	2.67	0.60	<0.01
History of low platelet counts^b^	0.46	0.92	0.37	0.13
History of anemia^c^	0.08	0.00	0.10	1.00
History of lung disease^d^	0.46	0.80	0.38	0.13
History of low neutrophil counts^e^	1.77	4.05	1.29	0.03
Prior DMARD use, %	95.00	85.60	97.23	<0.01
Prior MTX use, %	87.89	71.87	91.68	<0.01
Concurrent conventional DMARD use, %				
MTX only	–	–	68.6	–
>1 DMARD (including MTX)	–	–	15.3	–
Leflunomide	–	–	6.5	–
Hydroxychloroquine	–	–	4.4	–
>1 DMARD (excluding MTX)	–	–	2.6	–
Sulfasalazine	–	–	1.5	–
Other DMARDs	–	–	1.2	–
Prescription of biologic approved before 2006, %^f^	65.10	64.00	65.36	0.50
Initiation of biologic approved for monotherapy, %	69.28	76.53	67.57	<0.01
Type of biologic initiated, %				
Anti-TNF	90.95	88.93	91.43	0.03^g^
Non-anti-TNF	9.05	11.07	8.57	
Concurrent prednisone, %	30.16	28.93	30.44	0.43
Prednisone dose, mean (SD), mg/day^h^	6.4 (4.3)	7.1 (4.9)	6.3 (4.1)	<0.01

*anti*-*TNF* anti-tumor necrosis factor agent, *Bio CMB* biologic in combination with a conventional DMARD, *Bio MT* biologic monotherapy, *CDAI* clinical disease activity index, *CVD* cardiovascular disease, *DMARD* disease-modifying antirheumatic drug, *mHAQ* modified Health Assessment Questionnaire, *MI* myocardial infarction, *MTX* methotrexate, *RA* rheumatoid arthritis, *RF* rheumatoid factor

^a^ *P* values are for comparisons between patients who initiated Bio MT vs Bio CMB

^b^Low platelets defined as platelets <100,000/mm^3^

^c^Anemia defined as hemoglobin <8 g/dL

^d^Lung disease uses comorbidity indicators that varied across versions: lung disease, pulmonary fibrosis, or interstitial lung disease

^e^Low neutrophils defined as <1,000/mm^3^

^f^After 2006, more biologics became available

^g^ *P* value assessed using Fisher’s exact test

^h^Mean (SD) prednisone dose calculated only from patients receiving prednisone with dose reported (Bio MT, *n* = 205; Bio CMB, *n* = 912)

### Discontinuation of Prior DMARDs

Ninety-five percent of the patients included in this analysis had previously received conventional DMARDs, with MTX being the most commonly prescribed (87.9 %). The remaining 5 % of patients were started on biologic agents without prior use of conventional DMARDs. Reasons for discontinuation of prior DMARDs were unavailable for approximately 50 % of patients.

**Fig. 1:**
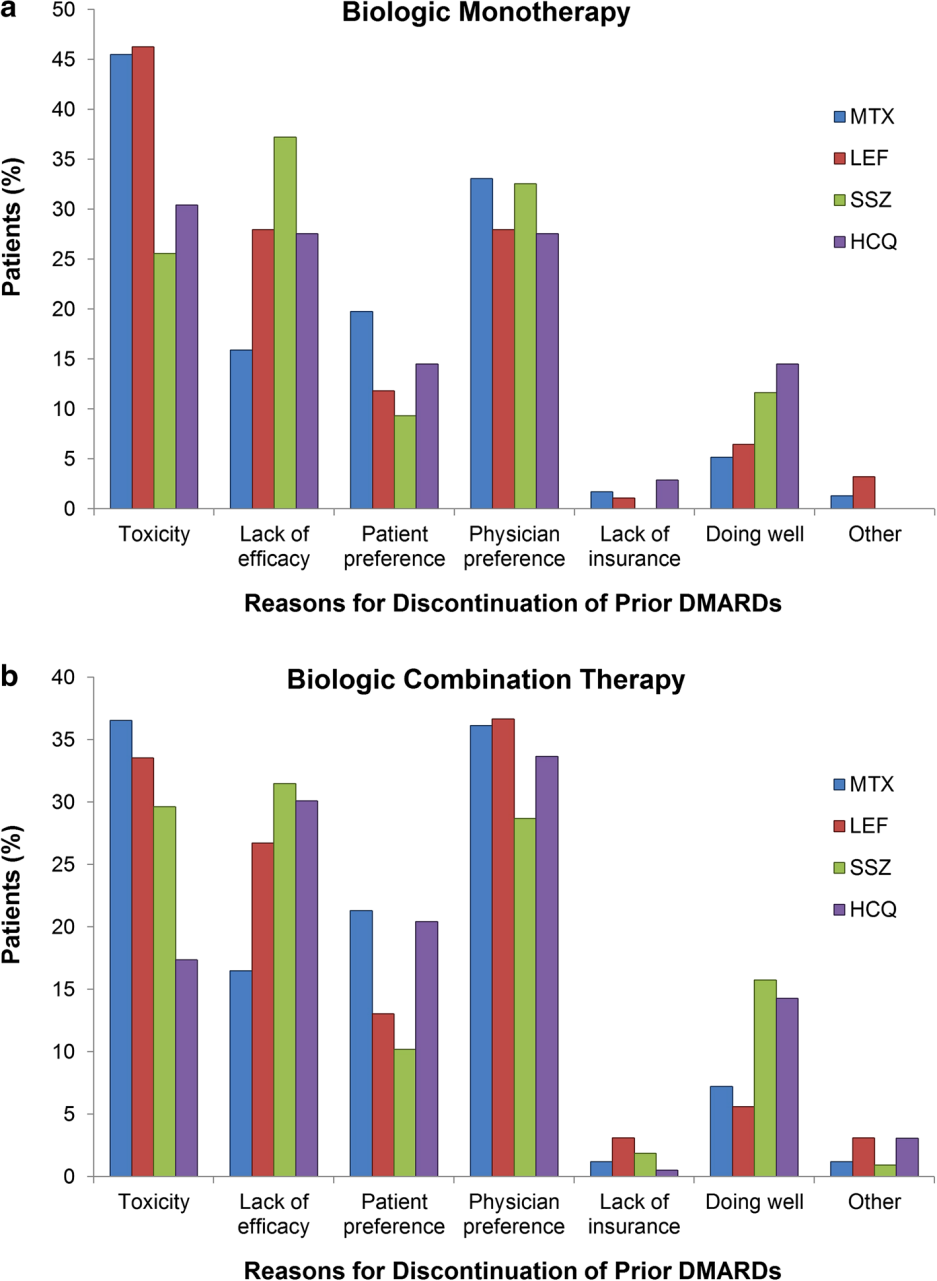
Reported reasons for discontinuation of prior DMARDs in biologic-naive patients initiating **a** Bio MT and **b** Bio CMB. *Bio CMB* biologic in combination with a conventional DMARD, *Bio MT* biologic monotherapy, *DMARD* disease-modifying antirheumatic drug, *HCQ* hydroxychloroquine, *LEF* leflunomide, *MTX* methotrexate, *SSZ* sulfasalazine

Of patients initiating Bio MT, the most common reasons for discontinuing any prior DMARDs were toxicity and lack of efficacy, with a significant proportion of discontinuations due to patient or physician preference (Fig. 1a). Furthermore, the most frequently reported reason for discontinuing prior MTX (45.5 %), leflunomide (46.2 %), and hydroxychloroquine (30.4 %) was toxicity, whereas the most frequently reported reason for discontinuing sulfasalazine was lack of efficacy (37.2 %).

Of patients who initiated Bio CMB, toxicity (36.6 %) was the most frequently reported reason for discontinuing prior MTX and lack of efficacy (31.5 %) was the most common reason for discontinuing prior sulfasalazine; however, reasons not related to toxicity or efficacy (e.g., physician preference) were the most common reasons for discontinuing leflunomide and hydroxychloroquine (Fig. 1b).

### Physician Characteristics in Prescribing Biologic Therapy in Biologic-Naive Patients

Physicians’ demographic and practice characteristics were available for 157 of 247 physicians (63.6 %) included in this analysis. The demographic characteristics of physicians at the time of prescribing biologic therapy to biologic-naive patients in this analysis are presented in Table 2. Of the 157 physicians included in the analysis with demographic information available, the majority were male, aged >50 years, had >19 years of experience and worked at private sites.

**Table 2:** Characteristics of physicians from Corrona prescribing biologic therapy in biologic-naive patients

	Physicians included in analysis
Total physicians, *N* ^a^	157
Female, %	35.0
Age, mean (SD), years	51.1 (9.2)
Years since training completed, mean (SD)	19.6 (10.3)
Years since graduation, mean (SD)	27.2 (10.8)
Site: private vs academic, %^b^	73.3
Region of United States, %^b^	
Northeast	36.0
Midwest	17.8
South	30.0
West	16.2

Physician characteristics refer to the time point of the initiation of the biologic agent

*SD* standard deviation

^a^Total number of physicians included in the analysis with demographic information available

^b^ *N* = 247; total number of physicians included in the analysis

### Predictors of Initiating Bio MT

Three mixed effects logistic regression models were fitted to estimate the odds for use of Bio MT in biologic-naive patients (Table 3). Model 1 (results presented in the second column of Table 3) represents the largest sample of biologic-naive patients and includes covariates such as history of comorbidities (hepatic disease and malignancy), swollen joint counts, whether the biologic initiated was approved for monotherapy in the United States, whether the treatment was initiated after 2006, and the impact of individual physician treatment decisions. History of hepatic events (OR 6.50; 95 % CI, 3.20–13.07), malignancies (OR 3.79; 95 % CI, 1.64–8.73), use of a biologic that was approved for monotherapy (OR 1.47; 95 % CI, 1.20–1.81), and variation in individual physician use of monotherapy (MOR 1.89; 95 % CI, 1.66–2.23) were all significantly associated with higher odds of monotherapy initiation.

**Table 3:** Adjusted odds ratios for biologic monotherapy vs combination in biologic-naive patients

Adjusted OR (95 % CI)	Model 1^a^ (*n* = 3,861)^b^	Model 2^a^ (*n* = 2,823)^b^	Model 3^a^ (*n* = 644)^b^
History of hepatic disease	6.50 (3.20–13.07)	7.49 (3.19–17.58)	5.20 (0.95–28.49)
History of malignancy	3.79 (1.64–8.73)	2.78 (1.02–7.59)	1.00 (0.19–5.40)
Swollen joint count	0.97 (0.95–0.99)	0.96 (0.95–0.98)	0.98 (0.94–1.02)
Use of biologic approved for MT	1.47 (1.20–1.81)	1.45 (1.13–1.86)	1.93 (1.08–3.43)
Initiated after 2006	0.83 (0.68–1.00)	0.79 (0.63–0.99)	–
Erosions	–	0.84 (0.68–1.03)	0.96 (0.62–1.49)
History of neutropenia	–	–	4.89 (1.16–20.59)
Random effect of individual physician’s treatment decisions	1.89 (1.66–2.23)	1.86 (1.61–2.25)	1.58 (1.23–2.72)

OR > 1 implies that monotherapy is more likely

*CI* confidence interval, *MT* monotherapy, *OR* odds ratio

^a^Three different models with various combinations of fixed effects from independent variables described above and a random effect of individual physician’s treatment decisions were fitted

^b^Models were fitted using available data among 3,923 previously biologic-naive patients initiating a biologic therapy

Models 2 and 3 (results presented in the third and fourth columns of Table 3, respectively) consider additional covariates, including the presence of erosions and history of neutropenia in addition to some or all covariates from model 1, but result in a reduced sample size. History of neutropenia (OR 4.89; 95 % CI, 1.16–20.59) was associated with biologic initiation as monotherapy in model 3.

As shown in Table 3, factors that influenced the likelihood of initiating Bio MT in all of the models included whether the biologic was approved for monotherapy at the time of prescription as well as the effect of variation in individual physician use of monotherapy. History of hepatic disease (models 1 and 2), history of malignancy (models 1 and 2), and neutropenia (model 3) also increased the odds of a patient being prescribed Bio MT in select analyses. Presence of erosions or whether therapy was prescribed before or after 2006 did not have an impact on the decision to initiate treatment as monotherapy in any of the 3 models.

## Discussion

Current European League Against Rheumatism (EULAR) and American College of Rheumatology guidelines for the management of RA emphasize that treatment should be a shared decision between physicians and patients, and should aim at reaching a target of low disease activity or remission [1, 2]. Treatment should begin with conventional DMARDs and, if there is no response, to initiate treatment with biologics in combination with conventional DMARDs [1, 2]. Importantly, the EULAR Task Force does not recommend use of biologics as monotherapy and strongly supports the use of all biologics in combination with MTX or other conventional DMARDs [2]. While studies with anti-TNFs have shown adalimumab and etanercept as monotherapy are comparable in efficacy to conventional DMARDs, using them in combination with conventional DMARDs is better than either treatment alone [5–7]. The EULAR Task Force mentions that if monotherapy must be started, then some supportive evidence for such a strategy exists only for tocilizumab [2].

The goal of this study was to describe the frequency of monotherapy biologic initiation in a real-world setting and to identify whether any factors beyond toxicities and intolerance to conventional DMARDs may influence the decision to start a biologic as monotherapy. In this US-based registry analysis, Bio MT was common and was initiated in approximately 1 of 5 biologic-naive patients with RA initiating a biologic agent. In previous biologics registry and claims database studies, 12–39 % of patients who were taking biologics did so as monotherapy [3, 10–16]. As expected, we identified that patients who received Bio MT frequently had prior toxicity to conventional DMARDs. Prior conventional DMARDs were also commonly discontinued due to lack of efficacy. However, reasons not related to either toxicity or efficacy, such as physician preference and patient preference, were frequently reported as reasons for discontinuing prior conventional DMARDs. In multivariate analyses, initiation of Bio MT in this biologic-naive population was associated with the presence of comorbidities, including history of hepatic disease, neutropenia, and malignancy.

In addition to the findings above, significant variation in physician use of monotherapy influenced the odds of initiating Bio MT in biologic-naive patients. This was assessed by calculating the estimated physician random effects, which measure the variation in rates of monotherapy among physicians due to unmeasured heterogeneity among physicians’ patient populations and in physicians’ treating patterns. A possible interpretation is that some physicians are more likely to start a biologic agent as monotherapy for reasons other than the ones recorded in the Corrona registry, which include toxicity, efficacy, cost and insurance-related reasons, or contraindication to conventional DMARDs. Some physicians may have prescription habits that differ from others or their patient populations may have characteristics that are difficult to objectively measure; these factors may be enough to lead to differential therapy decision making by treating rheumatologists.

This study analyzed initiations of only the first biologics for each participating patient; however, as RA is a chronic disease, treatment strategies are dynamic in nature and patients may have monotherapy treatment regimens prescribed intermittently alternating with combination regimens. The reasons patients initiate monotherapy with different biologics may vary and be biologic specific, including preference for a particular route or frequency of administration. Additionally, the patient-physician decision-making process often involves a complex dialog, and there may be reasons for changing from DMARDs to biologics that may not be possible to be fully described or captured.

## Conclusion

In conclusion, initiating biologic monotherapy in the biologic-naive population of this study was significantly influenced by variation in physician use of monotherapy, as well as whether the biologic was approved for monotherapy in the United States and history of hepatic disease, neutropenia, or malignancy. Further prospective analyses will follow prescription patterns to compare efficacy outcomes of patients initiating Bio MT with those initiating Bio CMB and evaluate whether Bio MT is associated with less toxicity.

## Electronic supplementary material

Below is the link to the electronic supplementary material.

Electronic supplementary material 1 (PPTX 115 kb)

## References

[1] Singh JA, Furst DE, Bharat A (2012). 2012 update of the 2008 American College of Rheumatology recommendations for the use of disease-modifying antirheumatic drugs and biologic agents in the treatment of rheumatoid arthritis. Arthritis Care Res.

[2] Smolen JS, Landewe R, Breedveld FC (2014). EULAR recommendations for the management of rheumatoid arthritis with synthetic and biological disease-modifying antirheumatic drugs: 2013 update. Ann Rheum Dis.

[3] Yazici Y, Shi N, John A (2008). Utilization of biologic agents in rheumatoid arthritis in the United States: analysis of prescribing patterns in 16,752 newly diagnosed patients and patients new to biologic therapy. Bull NYU Hosp Jt Dis.

[4] Gomez-Reino J (2012). Biologic monotherapy as initial treatment in patients with early rheumatoid arthritis. Rheumatology.

[5] Klareskog L, van der Heijde D, de Jager JP (2004). Therapeutic effect of the combination of etanercept and methotrexate compared with each treatment alone in patients with rheumatoid arthritis: double-blind randomised controlled trial. Lancet.

[6] Breedveld FC, Weisman MH, Kavanaugh AF (2006). The PREMIER study: a multicenter, randomized, double-blind clinical trial of combination therapy with adalimumab plus methotrexate versus methotrexate alone or adalimumab alone in patients with early, aggressive rheumatoid arthritis who had not had previous methotrexate treatment. Arthritis Rheum.

[7] van der Heijde D, Klareskog L, Rodriguez-Valverde V (2006). Comparison of etanercept and methotrexate, alone and combined, in the treatment of rheumatoid arthritis: two-year clinical and radiographic results from the TEMPO study, a double-blind, randomized trial. Arthritis Rheum.

[8] Kameda H, Kanbe K, Sato E (2011). Continuation of methotrexate resulted in better clinical and radiographic outcomes than discontinuation upon starting etanercept in patients with rheumatoid arthritis: 52-week results from the JESMR study. J Rheumatol.

[9] Dougados M, Kissel K, Sheeran T (2013). Adding tocilizumab or switching to tocilizumab monotherapy in methotrexate inadequate responders: 24-week symptomatic and structural results of a 2-year randomised controlled strategy trial in rheumatoid arthritis (ACT-RAY). Ann Rheum Dis.

[10] Heiberg MS, Koldingsnes W, Mikkelsen K (2008). The comparative one-year performance of anti-tumor necrosis factor alpha drugs in patients with rheumatoid arthritis, psoriatic arthritis, and ankylosing spondylitis: results from a longitudinal, observational, multicenter study. Arthritis Rheum.

[11] Soliman MM, Ashcroft DM, Watson KD (2011). Impact of concomitant use of DMARDs on the persistence with anti-TNF therapies in patients with rheumatoid arthritis: results from the British Society for Rheumatology Biologics Register. Ann Rheum Dis.

[12] Listing J, Strangfeld A, Rau R (2006). Clinical and functional remission: even though biologics are superior to conventional DMARDs overall success rates remain low–results from RABBIT, the German biologics register. Arthritis Res Ther.

[13] Askling J, Fored CM, Brandt L (2007). Time-dependent increase in risk of hospitalisation with infection among Swedish RA patients treated with TNF antagonists. Ann Rheum Dis.

[14] Mariette X, Gottenberg JE, Ravaud P, Combe B (2011). Registries in rheumatoid arthritis and autoimmune diseases: data from the French registries. Rheumatology.

[15] Lee SJ, Chang H, Yazici Y, Greenberg JD, Kremer JM, Kavanaugh A (2009). Utilization trends of tumor necrosis factor inhibitors among patients with rheumatoid arthritis in a United States observational cohort study. J Rheumatol.

[16] Sarzi-Puttini P, Antivalle M, Marchesoni A (2008). Efficacy and safety of anti-TNF agents in the Lombardy rheumatoid arthritis network (LORHEN). Reumatismo..

[17] Kaufmann J, Feist E, Roske AE, Schmidt WA (2013). Monotherapy with tocilizumab or TNF-alpha inhibitors in patients with rheumatoid arthritis: efficacy, treatment satisfaction, and persistence in routine clinical practice. Clin Rheumatol.

[18] Kremer JM (2005). The CORRONA database. Clin Exp Rheumatol.

[19] Atkinson JB, Swift LL, Lankford PG, LeQuire VS (1980). A generalized membrane defect in heritable myotonia: studies of erythrocytes in an animal model and patients. Proc Soc Exp Biol Med.

[20] Farrell FE. Chapter 9: Overview of maximum likelihood estimation. Regression modeling strategies: with applications to linear models, logistic regression and survival analysis. New York: Springer, 2001. p. 202–203.

[21] Larsen K, Petersen JH, Budtz-Jorgensen E, Endahl L (2000). Interpreting parameters in the logistic regression model with random effects. Biometrics..

