# Supplementary material for: Characteristics Associated with Biologic Monotherapy Use in Biologic-Naive Patients with Rheumatoid Arthritis in a US Registry Population
Source: Rheumatol Ther. 2015 Jan 27;2(1):85–96. doi: 10.1007/s40744-015-0008-9 (PMC4883255; doi:10.1007/s40744-015-0008-9)
Supplement: Supplementary file 1 — Electronic supplementary material 1 (PPTX 115 kb) [file 40744_2015_8_MOESM1_ESM.pptx]

## Slide 1
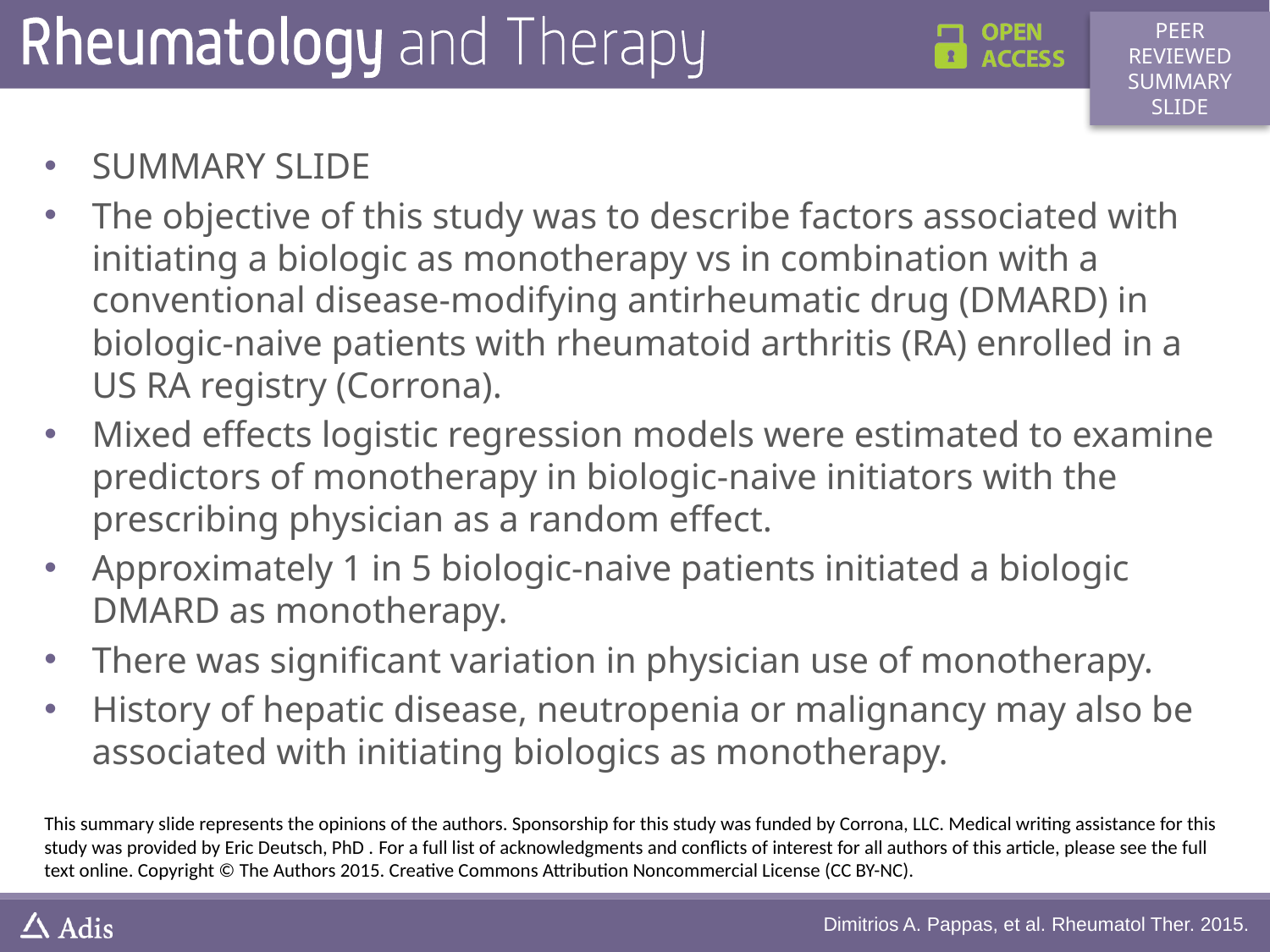

SUMMARY SLIDE
The objective of this study was to describe factors associated with initiating a biologic as monotherapy vs in combination with a conventional disease-modifying antirheumatic drug (DMARD) in biologic-naive patients with rheumatoid arthritis (RA) enrolled in a US RA registry (Corrona).
Mixed effects logistic regression models were estimated to examine predictors of monotherapy in biologic-naive initiators with the prescribing physician as a random effect.
Approximately 1 in 5 biologic-naive patients initiated a biologic DMARD as monotherapy.
There was significant variation in physician use of monotherapy.
History of hepatic disease, neutropenia or malignancy may also be associated with initiating biologics as monotherapy.
This summary slide represents the opinions of the authors. Sponsorship for this study was funded by Corrona, LLC. Medical writing assistance for this study was provided by Eric Deutsch, PhD . For a full list of acknowledgments and conflicts of interest for all authors of this article, please see the full text online. Copyright © The Authors 2015. Creative Commons Attribution Noncommercial License (CC BY-NC).
Dimitrios A. Pappas, et al. Rheumatol Ther. 2015.
